# Evaluating Type 1 Diabetes Resources to Improve Awareness and Knowledge of Type 1 Diabetes Within Community Sport Settings

**DOI:** 10.1002/edm2.70170

**Published:** 2026-03-03

**Authors:** Rachel J. Lim, Asha L. Parkinson, Heather C. Roby, Alison G. Roberts, Vinutha B. Shetty, Craig E. Taplin, Elizabeth A. Davis, Shaun Y. M. Teo

**Affiliations:** ^1^ Rio Tinto Children's Diabetes Centre The Kids Research Institute Australia, The University of Western Australia Perth Western Australia Australia; ^2^ School of Population Health Curtin University Perth Western Australia Australia; ^3^ Department of Endocrinology and Diabetes Perth Children's Hospital Perth Western Australia Australia; ^4^ Division of Paediatrics Within the Medical School The University of Western Australia Perth Western Australia Australia; ^5^ Centre for Child Health Research The University of Western Australia Perth Western Australia Australia; ^6^ School of Allied Health (Exercise Science) Murdoch University Perth Western Australia Australia

**Keywords:** community sport, confidence, education, knowledge, resources, type 1 diabetes

## Abstract

**Aim:**

A main challenge identified by youth during exercise and sport is the lack of knowledge and awareness around type 1 diabetes (T1D) particularly in community sport settings. Working with youth living with T1D, parents and community sport coaches, our team has developed resources for the T1D and sporting community. This study was to evaluate the acceptability and usability of the resources.

**Method:**

Participants completed an online evaluation survey consisting of a T1D quiz and ratings of subjective knowledge and confidence perception. The quiz on exercise and T1D management knowledge consisted of questions developed from the content of the resources and included: an understanding of T1D; signs and symptoms associated with T1D; identification and management of hypoglycaemic episodes. Participants were then provided access to the resources over a 4‐week exposure period. After 4 weeks, participants completed the same online survey. Participants were interviewed to collect qualitative information relating to the usability and acceptability of the resources, which included concepts around confidence, trust, and frequency of use.

**Results:**

24 coaches, mean age of 38 years, participated in the study. After the 4‐week exposure period, coaches' scores significantly better (*p* < 0.001) on the standardised T1D quiz from 67.3% to 81.8%. Similarly, coaches' self‐perceived ratings of knowledge and confidence increased significantly (*p* < 0.001) after the 4‐week period by an average of 1.6 Likert‐scale rating points across all four components. In interviews, coaches indicated high trust and perceived usefulness of the resources, and provided recommendations regarding further tailoring of information, language, and design of resources to enable wide community engagement.

**Conclusion:**

The resources developed were found to be informative, trustworthy, acceptable, and easy to use. Following minor amendments, the resources will be implemented into community sport settings through a nationwide launch.

## Graphical abstract

This study evaluated resources designed for community sport coaches, which aimed to increase awareness and knowledge of type 1 diabetes within community sport settings. The developed resources improved participating coaches' knowledge and confidence surrounding T1D management and exercise, and were perceived to be informative, trustworthy, acceptable, and easy to use.

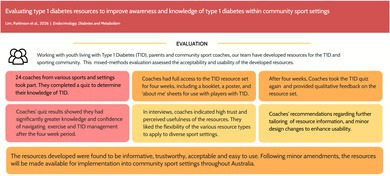

## 1 Introduction

It is established that one of the main challenges identified by youth during exercise and sport is the lack of knowledge and awareness around type 1 diabetes (T1D), particularly in community sport settings [1, 2]. While community sport is one of the most common settings in which youth perform exercise, a lack of educational exercise resources designed for community sport coaches has been identified and is associated with the perspective that they lack the knowledge, confidence, and understanding to provide adequate support for youth with T1D [3, 4]. There are a number of guidelines and consensus statements addressing exercise and T1D but these are generally aimed at health care professionals rather than community members without a health background [5]. Previous studies have shown that the delivery of T1D exercise education and programs can improve the knowledge and confidence of people living with T1D to help them manage their diabetes more safely and reduce hypoglycaemia risk, highlighting the importance of increasing T1D exercise‐related education to the community to better support those living with T1D [6, 7].

To address this gap, our previous research [4] involved conducting semi‐structured interviews with young people living with T1D, their parents, and community sport coaches to identify what they believe is important to include in educational resources for community sport settings. Additionally, we explored formats best suited for the T1D community and sport coaches, with the aim to increase awareness and support for young people living with T1D to participate safely in community sport [4]. Based on these findings, and upon consultation with an expert panel consisting of clinicians and allied health professionals specifically within the T1D field, a series of resources were developed. Content presented within the resources included: (i) what is T1D? (ii) signs and symptoms (iii) steps to treat hypoglycaemia (iv) treatment options, and (v) the effect of exercise on blood glucose levels (BGL). The content was produced in a variety of formats including (i) an A4 (21 × 29.7 cm) fact sheet (ii) an A3 (29.7 × 42 cm) poster (iii) a ‘My Diabetes’ handout (iv) a pocket wallet and (v) a booklet. All resources were designed to cater for different sports, settings and levels of engagement, each being identified as key factors during semi‐structured interviews. Using a participatory research design [8] to allow for further refinement of the educational resource, the developed resources were provided to a group of community members which included young people living with T1D and community sport coaches for feedback. This iterative process suggested improvements to the resources to ensure that they would be suitable and fit for purpose.

The overarching aim of this study was to explore the acceptability, feasibility and usability of the educational resources with our primary‐end users (i.e., community sport coaches) prior to resource implementation within the community. If they are found to be acceptable by community sport coaches, these resources will be implemented within the sporting community in Western Australia to inform sport coaches about exercise and T1D and better support young people living with T1D to engage in sport and exercise. These resources have the potential to be scaled across Australia and adapted to international settings.

## 2 Methodology

### 2.1 Study Design

A mixed methods study design was undertaken for the evaluation of the developed resources. Quantitative data was gathered through online surveys and qualitative data was collected through semi‐structured interviews. This study was approved by the Child and Adolescent Health Service Human Research Ethics Committee, Western Australia (RGS0000005933), and online e‐consent was obtained from all participants prior to participating in the study, which were generated and stored within REDCap [9, 10].

### 2.2 Participants

Purposive sampling of participant coaches was recruited from the wider sporting community within Western Australia through social media, website advertisements, and from the networks of the Department of Local Government, Sport and Cultural Industries. Coaches and volunteers across a range of different sports were recruited from metropolitan areas, with a range of coaching experience and educational backgrounds. Participants were excluded from the study if they: (i) had insufficient English language skills to give informed consent and complete the baseline and end of study surveys, or (ii) were unable to engage proficiently in an online interview.

### 2.3 Procedures

Eligible participants were invited to provide baseline data via an online survey including: (i) participant demographics (ii) a standardised T1D knowledge quiz, and (iii) ratings of subjective knowledge and confidence perception. For the demographic portion of the survey, participants provided responses regarding education, specific sport involvement, prior coaching of athletes living with T1D, and information relating to their coaching role such as employment type and duration. The knowledge quiz consisted of a series of “yes/no” and multiple‐choice questions (17 in total) to assess coaches' level of knowledge surrounding exercise and T1D management. These questions were derived specifically from the educational resource content developed in our previous research [4] along with input from an expert panel consisting of endocrinologists, diabetes educators, dietitians, and T1D researchers. The questions were used to assess the individual's knowledge around: (i) understanding of T1D (ii) signs and symptoms associated with living T1D, and (iii) identification and management of hypoglycaemic episodes. Additionally, four 5‐point Likert rating scale questions were used to assess subjective knowledge and perceived confidence surrounding exercise and T1D management, with a “0” response representing low level of knowledge/confidence and a “5” response representing high level of knowledge/confidence. The survey was reviewed by a consumer reference group for content and wording prior to its utilisation and took approximately 15 min to complete.

Upon completion of the online survey, coaches were mailed the educational resources to access for a 4‐week period. A follow‐up call was made by a member of the research team in the second week to ensure that participants had accessed the resources and to address any study related queries. Participants then completed the same questions from the baseline survey following the exposure period; the order of the questions and associated response options were randomised for each participant at baseline and end of study to reduce risk of recall bias in the survey responses. The online survey also included several open‐ended questions capturing participants' feedback on the resources. In addition to the online survey, participants were asked to complete a semi‐structured interview either via phone call or online via Microsoft Teams at the end of the study. The interview collected feedback relating to the usability and acceptability of the educational resources, which included concepts around confidence, trust, and frequency of educational resource use, such as: (i) *Was the resource easy/hard to understand?* (ii) *How often did you use the resource?* and (iii) *Did you trust the information and content?* The interviews also explored ideas relating to future use of the educational resources and target populations and settings where the resources would best be suited. All interviews were facilitated by research team members (1st author: RL 5th author: HR) experienced in qualitative research and conducting interviews within T1D research. All interview sessions were audio‐recorded for transcription. No field notes were taken during the interview and no interviews were repeated. All data collection tools are available in the Data S1.

### 2.4 Outcomes

Given the exploratory nature of the study and the use of a mixed methods design, the primary outcome was end user responses, both quantitative and qualitative, relating to the acceptability and usability of these educational resources.

### 2.5 Data Analysis

Descriptive statistics collected from the online surveys at baseline and end of study were calculated with mean (standard deviation (SD)) and percentages for continuous and categorical data, respectively. To determine if parametric data analyses were appropriate, descriptive data were subjected to normality tests (Shapiro–Wilk). Additionally, the magnitude of change for the quantitative data obtained from the online survey across the baseline and end of study was reported using Cohen's d (95% confidence interval) and interpreted as small (*d* = 0.2), moderate (*d* = 0.5), or large (*d* = 0.8) [11]. Data was downloaded from REDCap and analysed in JASP (Version 0.19.0) [Computer Software]. Statistical significance was set at *p* < 0.05.

Two authors (RL and AP) transcribed the audio‐recorded interviews and cross‐checked each transcript for accuracy. Transcripts were not returned to participants for comment. Data analysis was undertaken after the completion of all participant interviews and continued until no new codes emerged. All data was entered into NVivo software (QSR International). Two experienced researchers (RL and AP) used qualitative content analysis to analyse the data separately, inductively coding each transcript for manifest content [12]. Initial open coding was used to identify meaningful units of text which were commonly several sentences long. Units of text were organised under inductively developed codes, which were then grouped into sub‐categories and main categories reflecting resource feedback, experiences using the resources, and perceived outcomes of resource use [13]. Trustworthiness of the analysis process was promoted through note keeping and creating mind maps to demonstrate iterative development of the coding scheme, which assisted in sub‐category development [14]. All data are reported in line with the consolidated criteria for reporting qualitative research guidelines [15].

## 3 Results

Twenty‐four sport coaches (13 females and 11 males) with a mean age of 38.1 (12.6) years were recruited into the study. Of the 24 participants, one did not complete the end of study survey, and another did not complete the final interview as they could not be contacted. Of participating coaches, 75% were volunteers (*n* = 18) with the remaining 25% being paid coaches (part‐time: *n* = 5; full‐time: *n* = 1). In relation to the level of coaching training, 79% (*n* = 19) had previously completed some form of general coaching training, and 21% (*n* = 5) reported having previously received specific training regarding the management of T1D. Furthermore, 42% (*n* = 10) of the coaches had previously coached a person living with T1D, with 20% (*n* = 2) of these coaches having previously received some form of T1D training, either through the community or family members. A summary of the participant demographic characteristics along with the type of sports the participating coaches are involved with is presented in Table 1.

**TABLE 1:** Participant demographic.

Total (*n*)	24
Females/Males (*n*)	13/11
Age (years)	38.1 ± 12.6
Highest level of education attained (*n*)	
Secondary	6
Bachelor's Degree with Honours	5
Bachelor's Degree	4
Master's Degree	4
Certificate III & IV	3
Advanced Diploma and Diploma	1
Graduate Diploma	1
Type of sport coached (*n*)	
Soccer	6
Basketball	6
Australian Rules Football	5
Netball	3
Athletics	2
Cricket	2
Indoor/Field Hockey	2
Swimming	2
Volleyball	2
Baseball	1
Callisthenics	1
Cheerleading	1
CrossFit	1
Kayaking	1
Rowing	1
Rugby	1
Squash	1
Nature of coaching role (*n*)	
Volunteering service	18
Part‐time job	5
Full‐time job	1
Duration of coaching experience (*n*)	
5 years or more	16
1–2 years	4
3–4 years	4
Previous coaching experience with a person living with T1D (*n*)	
No	14
Yes (Average number of persons living with T1D coached)	10 (2)

### 3.1 Online Quiz and Perception Responses Analysis

Of the 23 coaches who completed both baseline and end of study surveys, the average correct score for the standardised 17‐item T1D quiz at baseline was 67.3 (14.6)%. After the 4‐week resource period, the average correct score percentage observed a significant improvement (*p* < 0.001) of 14.6 (16.3)% to 81.8 (12.8)% (Table 2). Additionally, all four self‐assessed knowledge and confidence ratings surrounding exercise and T1D management significantly increased (all *p* < 0.001) at the end of the study when compared to baseline ratings (Table 2).

**TABLE 2:** Changes in standardised T1D quiz and perceived knowledge and confidence ratings.

	Baseline	End of study	Change effect size	*p*‐value
Standardised T1D quiz (%)	67.3 (14.6)	81.8 (12.8)	14.6 ± 16.3; 1.1 (0.7, 1.5)	< 0.001
Knowledge and Confidence 1–5 Likert Scale Ratings				
Q1. How would you rate your knowledge on managing children with type 1 diabetes during sport and exercise?	2.3 (1.3)	3.9 (0.8)	1.6 ± 1.0; 1.5 (1.2, 1.8)	< 0.001
Q2. How would you rate your confidence in managing children with type 1 diabetes during sport and exercise?	2.5 (1.3)	4.0 (0.8)	1.5 ± 1.7; 1.4 (1.1, 1.7)	< 0.001
Q3. How would you rate your knowledge on how to treat children with type 1 diabetes during hypo and hyperglycaemic events?	2.0 (1.2)	3.7 (0.9)	1.7 ± 1.1; 1.8 (1.5, 2.0)	< 0.001
Q4. How would you rate your confidence in managing type 1 diabetes emergencies (severe hypo and hyperglycaemia)?	2.0 (1.2)	3.6 (1.0)	1.6 ± 1.1; 1.5 (1.2, 1.8)	< 0.001

*Note:* Data is presented as mean (SD). Effect size is presented as Cohen's d (95% CI).

### 3.2 Content Analysis

Structured interviews were conducted with 23 participants. Qualitative content analysis identified four key categories: (i) resource format, (ii) resource content, (iii) resource use, and (iv) current practices and implementation landscape. For each category, a description and list of sub‐categories is shown in Table 3.

**TABLE 3:** Categories and Subcategories within the Content Analysis.

Categories	Description	Subcategories
Feedback on Resource Format	Feedback related to the different formats provided within the resource collection	Tailored nature of resources Booklet Pocket Wallet A4 Fact Sheet Poster My Diabetes Handout
Feedback on Resource Content	Feedback related to the acceptability and relevance of informational content across the resource set	High trustworthiness of resource content Provides information to respond to a medical emergency Provides information to understand and enact coaches' responsibilities Need for streamlined resources to reduce superfluous information
Outcomes of Resource Use	Participant reported outcomes following the resource access period	Increased perceived knowledge of diabetes and exercise Increased awareness of importance of coaches' knowledge Increased confidence in communicating with children and families Lack of confidence despite increases in knowledge
Current Practices and Implementation Landscape	The landscape of the sporting environment, which provides practical considerations for resource implementation and engagement	Lack of awareness of physical health conditions Lack of training or resources Ad‐hoc nature of coaching Suggestions for dissemination and implementation

#### 3.2.1 Feedback on Resource Format

When describing what they liked about the resource set, eight coaches specifically mentioned the variety of resources provided, noting they worked well as complementary content, with each resource being useful in specific settings and scenarios. Other coaches noted that this variety would allow each coach to utilise the most feasible resources for their context, “I think that'll work for various people in various situations.” (P4). Two coaches noted that further tailoring of each resource to serve a specific purpose would help streamline the set. “I really loved the consistency of the information and how each document went into different depths, but I would say tailor each document for the purpose that it was going to by adjusting what exactly needs to be on there?” (P13). Specific feedback relating to acceptability, feasibility and perceived usability of each resource is outlined below.

##### 3.2.1.1 A4 Fact Sheet

Two coaches mentioned this resource as one of their favourites. Generally, feedback related to the amount and format of information on this resource, and five coaches commented that information should be reduced or simplified. In particular, several coaches noted that information related to medical devices was not necessary for this resource or their role as coaches and recommended streamlining the resource by removing this information. “I thought the pumps, devices and equipment… I don't think it's totally necessary because you're not, you know, anyone at the club is not going to be the one who delivers insulin to children.” (P9).

##### 3.2.1.2 Poster

While six coaches mentioned the poster as one of their favourites, overall feedback for this resource was mixed. Nine coaches noted positive feedback regarding the suitability of the design to being displayed in club rooms, first aid offices, or other training spaces, “That's the sort of thing that we could very, very easily see go up and on the wall in our equipment shed where all the coaches go and get equipment every week.” (P9). Coaches felt that displaying the posters would both help trainers revise their T1D knowledge and increase general diabetes awareness among all players.

In contrast, eight coaches noted that the quantity of information on the posters made it difficult to access key information, and this would hinder the use of the poster in club settings, “If I was to put that [poster] on my notice board at school. That's too wordy… So, more diagrams, more colour… Less on the page, that sort of gives them immediate information.” (P15). Six coaches suggested that the lack of a regular training space meant posters were not an ideal resource form for their club. Others noted that they conducted their training at centres or ovals with external management, which led to minimal opportunities to display posters, “We're one team or club essentially at a larger centre… So, we might be put on you know a different court each week so and yeah, and you know that's controlled by the stadium.” (P22).

##### 3.2.1.3 My Diabetes Handout

Nine coaches reported the My Diabetes Handout was the most helpful resource in the set. Seven coaches noted that having parents and players fill out this resource would capture all the essential information for them as a coach, such as how T1D was experienced by the individual and their specific support needs, “I think it's actually a really good thing to have. If I had a kid with diabetes with type 1, I would definitely be wanting to know these sort of details.” (P20). Coaches spoke favourably about how the resource provided players and their families with autonomy in choosing the level of communication and involvement of the coach with the child's management during sport participation. “I know that if I was coaching a child with type one diabetes, that would be my go to, basically. And I think it's a really nice way of maybe making me familiar with what that person might need and they can choose my level of involvement.” (P17).

##### 3.2.1.4 Pocket Wallet

Six coaches mentioned the pocket wallet was one of their favourite resources. The coaches noted that the resource covered what they considered to be the most important information in a simple and concise way. One coach felt that the pocket wallet was too small and also impractical for their training context, “I was gonna keep it in my wallet. I couldn't have my wallet on me when I'm training these kids or coaching them anyway.” (P20). In contrast, nine coaches noted the small size and portability of this resource as a key benefit. “My favourite part was the mini pocket thing, because I actually kept that with me… I just think that that's really good to just put in any little bags I carry… just in case anything came about.” (P12).

##### 3.2.1.5 Resource Booklet

Four coaches reported the booklet was one of their favourite resources. Nine coaches noted the booklet was a key source of more detailed information that would be helpful for those unfamiliar with T1D, “I showed it to all my instructors and there was one instructor who had no prior knowledge at all of diabetes. So when she read the poster and the other pieces, she had a few questions. But then when she was able to read the full booklet, … that was very easily answered within the booklet.” (P5). In addition, several coaches felt that the depth of information provided in the booklet would allow the other resources to be further simplified to focus on essential information, as the booklet could be referred to for further details, “The booklet is where all the nitty gritty, the nuts and bolts is, that right? So, I feel as though the A4 fact sheet could be simpler, and you can always make reference to the book.” (P2).

#### 3.2.2 Feedback on Resource Content

Ten coaches provided positive feedback regarding the resources' content on the interaction between T1D and sport, signs and symptoms to look out for, and the list of precautionary measures to undertake before, during, and after training or games. This content was perceived to be the most helpful and relevant to regular responsibilities of the coach, “If it's a big training or a big game and they can't miss it and they're kind of ignoring some of their blood sugar things, it's kind of your responsibility as a coach. You should know…some of their signs that they're having a blood sugar event.” (P3).

Coaches felt that information on the impact of exercise on BGL raised their awareness of the complexities of managing T1D, especially in sports with fluctuating levels of strenuousness. Four coaches noted that the resources clearly stated what tasks came under their responsibility as a coach and provided practical strategies for supporting players living with T1D, which made their role as a coach more transparent, “I think specifically like the precautionary measures for exercise was just a simple, small thing that I can apply.” (P13). Eight coaches reported positive feedback on the usefulness of the resources to help them identify and respond to acute medical situations, “Especially if you've got coaches who respond differently in emergency situations, it's kind of a very helpful piece of information to remind myself of what I need to do.” (P17).

While many coaches felt that all necessary information was covered, six coaches felt that across the resources, it was difficult to quickly identify the essential information that would be useful in a medical situation, “The information was a little much to be looking at and if we've got something happening, I think it's better if it's clearer and a little more, you know, concise.” (P14). Coaches recommended that information related to signs, symptoms, and treatment should more clearly stand out on each resource except for the booklet, which was least likely to be used in an emergency.

Regarding trustworthiness, 18 coaches noted strong trust in the content provided within the resources. In discussing the factors contributing to their trust, 12 coaches noted that the organisation affiliations made them confident that the information was provided by a reputable source, “It's coming from a good source. So Perth Children's Hospital obviously is the main source of paediatric diabetes management.” (P24). Three coaches noted that the use of a solid evidence base and stakeholder consultation process to develop the resources contributed to their trust, while an additional three stated that their trust came from the logical and straightforward way the information was set out within the resources.

#### 3.2.3 Outcomes of Resource Use

Aligning with the changes in quantitative scores following resource use, the overwhelming majority of coaches felt that the resources increased their knowledge of T1D, how it interacts with exercise, and what diabetes signs and symptoms they may encounter, “I think I'd be very confident—if someone told me that they had diabetes—to ask for their plan and to know what symptoms to look for… I would never have known that sort of stuff before.” (P16). Others noted that although they had existing knowledge regarding how exercise may impact diabetes management, they now had increased knowledge of responding to BGL episodes. “I think my knowledge around sort of what to do in certain situations definitely improved… I had knowledge about what certain exercises could do to blood sugar levels and that sort of thing, but I really didn't have much in terms of, you know, what should I do in those situations?” (P21).

Ten coaches reported that the resources increased their confidence in initiating T1D‐related conversations with players and their families, as well as engaging in proactive planning with families regarding their role as a coach in supporting players' management. For example, one coach described a prior experience in which a player was showing signs of hypoglycaemia after an intense period of exercise, stating that the resources improved their capability to respond to similar situations in the future, “Whereas now I definitely feel like I would know to have that conversation beforehand. And I would know some of the things that I could do to help keep her safe. That was the one thing that kind of stood out in my mind… that particular situation, I feel like I would handle better now with just with a bit more knowledge of the impact that exercise has and what needs to be done sort of afterwards.” (P17).

In contrast, three coaches noted that despite their increased knowledge, they did not yet feel confident in putting this into practice in an emergency scenario, preferring to refer to medical professionals or parents. “I feel I could provide advice on what to do, but if hypoglycaemia actually came about, I would probably go and like ask for a nurse… just to actually do the treatment. But like, I could identify symptoms and what's going on.” (P12).

#### 3.2.4 Current Practices and Implementation Landscape

Coaches reported that there was no training or resources regarding medical conditions provided to them through their organisation, “It's quite primitive really.” (P23). Ten coaches noted that while their organisation provided information on other conditions such as asthma, nothing was provided regarding supporting children living with T1D, “At my last swim school, there was no information given to me to assist with type one diabetes, except what was relayed from the parents.” (P5). A further eight coaches noted an absence of available information regarding medical conditions more broadly. Even when players were required to complete medical forms and report health conditions when joining the sports team, this information of individuals was often not provided to coaches “…We don't get that information, so I could be coaching someone who has diabetes… that I need to be aware of, and I'm not.” (P15).

Coaches explained that the ad‐hoc nature of coaching, including the various settings and contexts and the large role of volunteers, presented barriers to resource implementation. A key issue was the lack of clear communication lines and absence of protocols for disclosing and managing health conditions within sporting clubs, “There's no management plan in place by the club, there's nothing… It's not exactly amazing structure, realistically.” (P11). Compounding this issue, six coaches noted that many coaches were volunteers, casuals, or older youth players working informally, creating additional difficulties for information provision, “I guess when I do stand in, I should know this [information]… But it's often not the case when you're a volunteer, isn't it? You just get called on at the last minute.” (P18). Finally, two coaches noted that players' fluctuating attendance was common in their sport, which would present challenges for coaches' ability to identify children living with T1D as well as access their medical information, “So I suppose there's a bit of a loophole and a bit of a gap in that case, when you don't also know who's coming… kids rock up different days, different times, you rarely get the same children…” (P15).

The coaches suggested several implementation strategies in consideration of these challenges. Nine coaches suggested that having the resources distributed in the current format by overarching state bodies for each sport was an optimal strategy, as it would allow broad reach and dissemination, “I think if the information comes out of the state body that manages clubs… then it's going to get distributed among the clubs and among the people that need to know. And you'd find it then, it would then be spread among all the clubs in Western Australia.” (P14). Four coaches suggested that adapting the resources into an educational course would allow integration with popular existing e‐courses delivered by overarching bodies such as the Australian Sporting Commission. Finally, three coaches suggested dissemination of the resources through online sports team management applications for coaches and players, as these allow information to be provided instantly to all members of their association.

## 4 Discussion

Our present study aimed to address the identified gap within the T1D community relating to the lack of available educational exercise resources for community sport across Western Australia. Our study explored the acceptability, feasibility, and usability of a series of co‐designed educational resources with community sport coaches in Western Australia to help provide essential information relating to its potential implementation within the community. Overall, both the quantitative and qualitative data collected during the study suggest a favourable and positive response towards the acceptability and usability of the co‐designed educational resources by community sport coaches in Western Australia.

In our cohort of sport coaches, access to the resources resulted in a moderate improvement (*d* = 0.4) in exercise knowledge. Similar results have been shown in other studies using pre‐ and post‐assessments studies to assess a change in knowledge in youth coaches provided with concussion educational program interventions [16, 17, 18]. Parker et al. [18] investigated the change in coaches' knowledge following an online concussion course and found a 37.4% improvement in coaches' ability to select the correct concussion response strategy to decrease potential health risks. This is comparable to the need for coaches to respond to risks associated with hyperglycaemia and hypoglycaemia when players living with T1D take part in physical activity. In our study, we observed a significant 17% and 63% improvement in sports coaches' knowledge relating to signs and symptoms of hyperglycaemia and hypoglycaemia, respectively. Importantly, results from our previous work highlighted that coaches, young people living with T1D and their families considered that signs and symptoms of hyperglycaemia and hypoglycaemia to be the most important topic to be included in any developed educational resources [4]. Hence, the results of our present study highlight the relevance and usefulness of the designed resources. We also observed similar results in coaches' perceived ratings of knowledge and confidence around managing T1D and dealing with T1D emergencies, with a moderate increase in perceived ratings across all four criterions being reported. Similarly, Glang et al. [19] evaluated the ACTive e‐learning program for community coaches in sport concussion prevention and management practices and found that this program improved coaches' general knowledge of concussion by 41%, recognition of symptoms by 37.5%, and coaches reported an increase in confidence. Of note, the coaches in Glang et al.'s [19] study had access to the program for 15–20 min and the post‐test was conducted immediately after. Therefore, recency bias may be a contributing factor to the improvement in scores. In contrast, the 4‐week exposure period in our present study suggests that participating coaches may have had sustained improvements in knowledge and confidence.

The significant improvement in the T1D quiz scores suggests that the resources were a beneficial educational tool, which subsequently translated into significant increases across perceived knowledge and confidence of coaches relating to the management of T1D during sport. Importantly, Kerr et al. [20] reported that coaches who had completed educational training resulted in less head injuries compared to coaches who did not receive any formal educational training. Additionally, Kroshaus et al. [17] found that the exposure to training resources increased communication intentions between youth and coaches which lead to increased safety during sport. This highlights how the use of resources and training tools can reduce the various risks that may arise during physical activity, which could potentially promote better outcomes for players, especially in youth athletes. Collectively, these observed improvements have the potential to allow sports coaches to provide better support during sport to their players living with T1D, which was the overarching aim of the resource development.

Qualitatively, coaches provided positive feedback regarding the various formats within the resource set, suggesting this would allow them the freedom to mix‐and‐match resources based on their preferences and needs. While the coaches identified potential improvements related to streamlining the resource content, the resources were perceived as a reputable and relevant source of information for coaches training individuals living with T1D. In line with improvements in quantitative scores, the coaches reported increases in their perceived knowledge as well as increased confidence in providing support to players managing T1D, suggesting the resources were an acceptable and feasible format to improve knowledge.

### 4.1 Strengths and Limitations

A key strength of our study is the inclusion of a team‐based approach to analysis and interpretation of data, whilst maintaining quality and reporting standards for content analysis in accordance with the consolidated criteria for reporting qualitative research guidelines [14]. Another strength was the diverse sample of participants in this study, which included community sports coaches from different sports and coaching roles. Furthermore, our complementary mixed methods approach strengthens the findings by validating quantitative and qualitative data to provide a more credible, applicable and meaningful interpretation of the data [21].

Given the exploratory nature of our study, a methodological limitation is the small sample size of participants, which was skewed towards volunteer coaches and those coaching within metropolitan regions. Consequently, to better investigate the translational impact of the developed educational resources within community sports, future research should recruit a larger sample size of participants with different demographics based on coaching experience, nature of coaching work, and geographical location.

In addition, our present study converted Likert‐scale responses into interval data during the data analysis process and presented them in the results section. We do acknowledge that this may be a minor limitation due to the potential for the introduction of assumptions around equal spacing between categorical responses. Despite this analytical approach being considered common and acceptable within similar study designs and that the Likert‐scale responses made up a small portion of the online survey used to assess the acceptability and usability of the developed resources, future studies may need to consider a more appropriate analytical approach to improve upon result presentation transparency.

Although not considered to be a limitation given the exploratory nature of the present study, we acknowledge that more robust performance‐based or behavioural outcome measures need to be incorporated in future study designs to better understand and assess the feasibility of the developed resources from an implementation perspective. Additionally, exploratory data obtained from pilot studies similar to our current study can be used to inform future studies, which should include evaluation tools validated for the specific population of interest, along with greater practical application significance.

## 5 Implications for Implementation and Future Research

A potential avenue for future research is to explore the applicability of the developed educational resources for youth with medical complexity or comorbid conditions. While evidence‐based recommendations are the primary source of information for developing educational resource content, the extent of evidence and availability of educational resources relating to exercise/sport performance for children and youth with complex co‐morbidities is less than for those with mild to moderate conditions [22]. While our study demonstrates the suitability of the resources in the context of children living primarily with T1D, the inclusion of diverse samples in future studies will promote the development of robust educational resources to include appropriate content tailored towards persons living with T1D who are living with comorbidities.

This study revealed important considerations for implementing educational resources within sporting clubs. Coaches noted several contextual factors contributing to implementation challenges, including the ad‐hoc nature of coaching, lack of protocol and outlined processes, and overarching club culture. This aligns with prior research demonstrating that the environment of the sporting club, the expectation and influence of players' parents, and practical constraints all impact coaches' abilities to integrate their knowledge into practice [23]. Devising appropriate implementation strategies for this context is necessary for resource uptake, with several strategies suggested from the participating coaches, including dissemination by overarching sporting bodies or through mandatory educational courses. Moreover, rather than finding a ‘one‐size‐fits‐all’ solution, it is likely that successful translation will necessitate devising various implementation approaches which can be tailored to the specific contexts faced by each sport, organisation or club. For example, organisations that are primarily staffed by volunteers or face high coach turnover rates are less likely to benefit from implementation via mandatory educational courses than organisations that are staffed by ongoing, paid coaches. Furthermore, appropriate use of the resources is pre‐meditated on the ability of each sporting organisation to both identify players with living T1D and to provide this information to the relevant coaches. Where this is lacking, successful implementation will depend on integration of the existing resources with additional policies for onboarding players living with T1D. Future research could explore the effectiveness of potential strategies through implementation trials at various levels, including dissemination at state‐level, within individual clubs, and integration within organisational policy and procedures.

The cultural context within which the resources were designed and evaluated also has implications for the implementation of the resources in diverse settings. The Australian Government provides all children living with T1D with fully subsidised continuous glucose monitoring (CGM) until the age of 21, and the resources were designed with this context in mind. Globally, access to this diabetes technology is piecemeal and highly variable, shaped by individual contexts including ethnicity and insurance coverage, as well as national contexts such as a country's geography and national income [24]. As such, the developed resources are likely to lack applicability to settings with low access to diabetes technologies. As the successful implementation of the resources relies on first identifying players living with T1D, the willingness of players to disclose their T1D to their coaches or organisations is also worth considering. Culture can be a salient factor shaping how individuals living with T1D and their families manage diabetes, and several cultures may abide by customs that involve little to no disclosure of T1D to those outside the family [25]. As such, the developed resources are likely to lack relevance to coaches working within cultures or settings in which talking about T1D is not the norm.

## 6 Conclusion

Through this exploratory evaluation of co‐designed T1D educational resources for community sport settings, the content of the resources allowed for improvements in participants' knowledge and confidence regarding the management and treatment of T1D after a 4‐week exposure period. The resources were found to be informative, trustworthy, acceptable, and easy to use. The results from this study have provided valuable information relating to the translation and implementation of educational resources in the wider sporting community. Future research should focus on evaluating the long‐term efficacy of these educational resources on sporting participation outcomes of persons living with T1D and exploring implementation strategies for the resources, as well as the scalability of the resources at a national or international level.

## Author Contributions

**Rachel J. Lim:** methodology, investigation, data curation, formal analysis, writing – original draft, writing – review and editing, funding acquisition. **Asha L. Parkinson:** data curation, formal analysis, writing – original draft, writing – review and editing. **Heather C. Roby:** conceptualisation, methodology, investigation, writing – review and editing, project administration, funding acquisition. **Alison G. Roberts:** conceptualisation, methodology, data curation, funding acquisition. **Vinutha B. Shetty:** conceptualisation, methodology, writing – review and editing, funding acquisition. **Craig E. Taplin:** conceptualisation, methodology, writing – review and editing, supervision. **Elizabeth A. Davis:** conceptualisation, writing – review and editing, funding acquisition. **Shaun Y. M. Teo:** conceptualisation, methodology, data curation, formal analysis, writing – original draft, writing – review and editing, supervision, funding acquisition.

## Funding

The author(s) declare financial support was received for the research, authorship, and/or publication of this article. This study was supported by the general grant funding provided by the Diabetes Australia Research Program (Y23G‐TEOS). The study team was supported by Breakthrough T1D Australia (grant number 5‐SRA‐2021‐1088‐M‐X).

## Ethics Statement

Informed consent was obtained from all participants involved in the study. The studies involving humans were approved by Child and Adolescent Health Service Human Research Ethics Committee, Western Australia (RGS0000005933). The studies were conducted in accordance with the local legislation and institutional requirements. Informed consent was obtained from all participants involved in the study.

## Conflicts of Interest

The authors declare no conflicts of interest.

## Supporting information


**Data S1:** edm270170‐sup‐0001‐supinfo.docx.

## Data Availability

The raw data supporting the conclusions of this article will be made available by the authors, without undue reservation.
