# Supplementary material for: Evaluating Type 1 Diabetes Resources to Improve Awareness and Knowledge of Type 1 Diabetes Within Community Sport Settings
Source: Endocrinol Diabetes Metab. 2026 Mar 3;9(2):e70170. doi: 10.1002/edm2.70170 (PMC12957531; doi:10.1002/edm2.70170)
Supplement: Supplementary file 1 — Data S1: edm270170‐sup‐0001‐supinfo.docx. [file EDM2-9-e70170-s001.docx]

**SUPPLEMENTARY MATERIALS**

**QR Code for Resource Access**

**
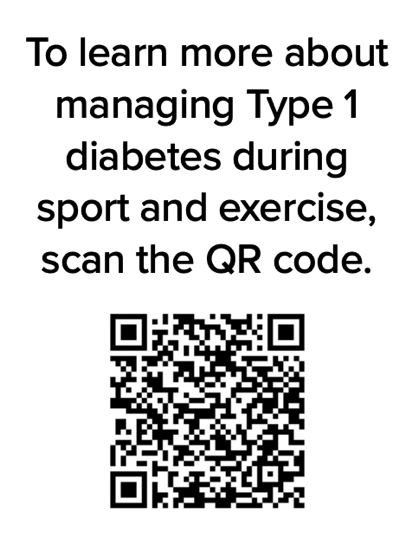
**

**Participant online survey (pre-evaluation)**

| **Participant Details & Information** | | | | | | |
| --- | --- | --- | --- | --- | --- | --- |
|  | | |  | | | |
| Age (in years): | | | ________________________ | | | |
|  | | |  | | | |
| Gender: | | | □ Male | | | |
|  |  |  | □ Female | | | |
|  |  |  | □ Other | | | |
|  |  |  | □ Prefer not to say | | | |
|  | | |  | | | |
| What is the highest level of education you have attained? | | | □ Secondary education _(Year 9 and below)_ | | | |
|  |  |  | □ Certificate I & II level | | | |
|  |  |  | □ Secondary education _(Year 10 and above)_ | | | |
|  |  |  | □ Certificate III & IV level | | | |
|  |  |  | □ Advanced diploma and diploma level | | | |
|  |  |  | □ Graduate diploma and certificate level | | | |
|  |  |  | □ Bachelor degree level | | | |
|  |  |  | □ Postgraduate degree level | | | |
|  |  |  | □ Master degree level | | | |
|  |  |  | □ Doctoral degree level | | | |
|  | | |  | | | |
| What sport/s do you coach/are you involved in? | | | Sport 1: _________________ | | | |
|  |  |  | Sport 2: _________________ | | | |
|  |  |  | Sport 3: _________________ | | | |
|  |  |  | Sport 4: _________________ | | | |
|  |  |  | Sport 5: _________________ | | | |
|  | | |  | | | |
| Is coaching your sport/being involved in your sport a: | | | □ Full-time job | | | |
|  |  |  | □ Part-time job | | | |
|  |  |  | □ Casual employment | | | |
|  |  |  | □ Volunteering service | | | |
|  |  |  | □ Practicum placement | | | |
|  | | |  | | | |
| How long have you been coaching/involved in your sport? | | | □ Less than 1 year | | | |
|  |  |  | □ 1-2 years | | | |
|  |  |  | □ 3-4 years | | | |
|  |  |  | □ 5 or more years | | | |
|  | | |  | | | |
| Have you coached an individual who is living with type 1 diabetes? | | | □ Yes  *# of individuals:* ___________ | | | |
|  |  |  | □ No | | | |
|  | | |  | | | |
| Have you received any general coaching training? | | | □ Yes  *Institute/Organisation:* ___________ | | | |
|  |  |  | □ No | | | |
|  | | |  | | | |
| Have you received any training to coach in your sport/sports specifically? | | | □ Yes  *Institute/Organisation:* ___________ | | | |
|  |  |  | □ No | | | |
|  | | |  | | | |
|  | | | | | | |
| **Knowledge and Confidence Perceived Ratings** | | | | | | |
|  | | | | |  | |
| How would you rate your… | | | | | | |
|  | | | | | | |
| ***Knowledge*** *on managing children with type 1 diabetes during sport and exercise?* | | | | | | |
|  | | | | | | |
| Low | Fair | Reasonable | | Good | | High |
| ○ | ○ | ○ | | ○ | | ○ |
|  | | | | |  | |
| ***Confidence*** *in managing children with type 1 diabetes during sport and exercise?* | | | | | | |
|  | | | | | | |
| Low | Fair | Reasonable | | Good | | High |
| ○ | ○ | ○ | | ○ | | ○ |
|  | | | | |  | |
| ***Knowledge*** on how to treat children with type 1 diabetes during hypo and hyperglycaemic events? | | | | | | |
|  | | | | | | |
| Low | Fair | Reasonable | | Good | | High |
| ○ | ○ | ○ | | ○ | | ○ |
|  | | | | |  | |
| ***Confidence*** in managing type 1 diabetes emergencies (severe hypo and hyperglycaemia)? | | | | | | |
|  | | | | | | |
| Low | Fair | Reasonable | | Good | | High |
| ○ | ○ | ○ | | ○ | | ○ |
|  | | | | | | |

**Type 1 diabetes and exercise quiz**

|  |  | |
| --- | --- | --- |
| **Type 1 Diabetes and Exercise Quiz** | | |
| **Which of the following devices show current glucose readings and trend arrows that provide information that is useful for the management of type 1 diabetes?** | | |
| □ (a) Insulin pump | | |
| □ (b) Continuous glucose monitor | | |
| □ (c) Insulin pen | | |
| □ (d) Ketone meter | | |
|  |  | |
| **TRUE OR FALSE: A generic management plan will suffice for the management of Type 1 Diabetes during sport and exercise, and an individual management plan is not necessary.** | | |
| □ True | | |
| □ False | | |
|  |  | |
| **TRUE OR FALSE: Type 1 diabetes is caused by the body not using insulin as well as it should, thereby developing insulin resistance.** | | |
| □ True | | |
| □ False | | |
|  |  | |
| **Hyperglycaemia is a blood glucose level of ___________ :** | | |
| □ (a) More than 5 mmol/L | | |
| □ (b) More than 10 mmol/L | | |
| □ (c) More than 15 mmol/L | | |
| □ (d) More than 20 mmol/L | | |
|  |  | |
| **Hypoglycaemia is a blood glucose level of ___________ :** | | |
| □ (a) Less than 4 mmol/L | | |
| □ (b) Less than 5 mmol/L | | |
| □ (c) Less than 6 mmol/L | | |
| □ (d) Less than 7 mmol/L | | |
|  |  | |
| **Which of these are not a useful as an immediate hypoglycaemia treatment option?** | | |
| □ (a) 6 to 7 jellybeans | | |
| □ (b)100 ml of full-strength soft drink | | |
| □ (c) 150 to 200 ml of fruit juice | | |
| □ (d) 2 to 3 pieces of dried fruits | | |
| Is the following **sign or symptom** associated with hyperglycaemia (high glucose levels)? | | |
|  | | |
|  | Yes | No |
| *Excessive thirst* | ○ | ○ |
| *Weakness, trembling or shaking* | ○ | ○ |
| *Change of behaviour (usually irritable)* | ○ | ○ |
| *Hunger* | ○ | ○ |
| *Frequent urination* | ○ | ○ |
|  |  |  |
| Is the following **sign or symptom** associated with hypoglycaemia (low glucose levels)? | | |
|  | | |
|  | Yes | No |
| *Light headedness/headache* | ○ | ○ |
| *Lack of concentration* | ○ | ○ |
| *Numbness around the lips/fingers* | ○ | ○ |
| *Lethargy* | ○ | ○ |
| *Blurred vision* | ○ | ○ |
|  |  | |
|  |  | |
| **In ______________, the immune system attacks the cells in our pancreas that produce insulin, called beta cells. Without the insulin, glucose can build up in the bloodstream in life-threatening amounts.** | | |
| □ (a) Latent autoimmune diabetes of adults (LADA) | | |
| □ (b) Type 2 diabetes | | |
| □ (c) Gestational diabetes | | |
| □ (d) Type 1 diabetes | | |
|  |  | |

**Open ended online questions**

| **Online: Open-ended Questions** | | | |
| --- | --- | --- | --- |
| During the last 4 weeks when you had access to the resources, how often did you use it?  _(_*_Please provide a brief explanation for i) why you used it frequently/occasionally) and ii) which resources did you access)_* | | | |
|  | | | |
|  |  | |  |
| On a scale of 1 to 5, with 1: Very easy and 5: Very difficult, was the resource content easy/difficult to understand? *_(Please provide a rating and an explanation for the rating)_* | | | |
|  | | | |
|  |  | |  |
| On a scale of 1 to 5, with 1: Not at all and 5: Definitely, did you feel you could trust the information that was provided to you through the resources? *_(Please provide a rating and an explanation for the rating)_* | | | |
|  | | | |
|  |  | |  |
| On a scale of 1 to 5, with 1: Not at all and 5: Definitely, did the information you gained through the resources improve your level of knowledge surrounding exercise and type 1 diabetes management? *_(Please provide a rating and an explanation for the rating)_* | | | |
|  | | | |
|  |  | |  |
| On a scale of 1 to 5, with 1: Not confident at all and 5: Very confident, how confident were you in applying the knowledge gained from the resources into your respective sporting community?  *_(Please provide a rating and an explanation for the rating)_* | | | |
|  | | | |
|  |  | |  |
| Was there anything about the resource content/design or specific components that you think can be improved? If so, please provide us with examples and suggestions. | | | |
|  | | | |
|  |  | |  |
| Overall, on a scale of 1 to 5, with 1: Not helpful at all and 5: Very helpful, how helpful was the resources in assisting you during your coaching practice?  *_(Please provide a rating and an explanation for the rating)_* | | | |
|  | | | |
|  |  | |  |
| Lastly, would you recommend these resources to others in the sporting community? If so, which group of individuals in the community do you think will benefit from these resources? | | | |
|  | | | |
|  |  | |  |
|  | |  | |

**\ Semi structured interview guide**

**Semi-structured Interview Guide**

The aim of the interview is to determine i) what the coaches have learned from the resources, ii) how they have disseminated this knowledge and iii) how they have used these resources within their sporting teams?

*Points to take note:*

- *Explain that all questions are valid and there are no ‘wrong’ answers.*
- *Remind participants of the importance of maintaining confidentiality of all information discussed during interview*
- *Explain that we want to know how useful the resources have been and how they have been used*

Thank you for agreeing to trial the resources for the last 4 weeks.

1. **Can you please describe what sport you coach, how many teams you have, do you have assistant coaches or volunteers that help you**?

- If you have assistants / volunteers – how many, how often are they around
- Do you coach anyone with T1D that you now of now / previously?
- Club or independent coach and to which club?

**2) Tell me about your diabetes knowledge…**

- Did you have any previous knowledge about T1D – if yes, please explain/elaborate?
- If no, was the information in the resources enough or did you have to research more?
- Was there already information regarding T1D at your club? If so, what was it – whom is it from?

1. **Thinking about the resources…**

- Tell me what you thought about the content, layout and colour
- Was there anything about the resource you think needs to be altered or changed?
- Did anyone else have comments about the resources?

1. **Tell me how the information helped you understand diabetes better**

- Did the resources improve your knowledge? If yes/no – please explain/elaborate
- Did you feel like you could trust the information in the resources? If yes/no – please explain/elaborate?
- How confident do you feel applying the learnt knowledge into your sporting community? And did you feel like you can remember the content?
- Did the information you gained from the resources improve you level of knowledge around exercise and T1D management? Explain.

1. **Can you tell me how you used the resources?**

- How often did you refer to or use them? Elaborate how and why.
- How did they help you with your coaching?
- Who in your team used them?
- Did you share them with parents or other children / adults?

Methods of dissemination through the club (how do they distribute information now and how to they think we can disseminate the information)

Postage cost (hard vs electronic copies)

**Lastly,**

1. **Do you think these resources would be helpful to others in the community, if so, who do you think can benefit from this?**

**a). Since I last spoke to you at the 2 week mark, how many more times have you accessed the resources in the last 2 weeks?**
